# Trends in mortality from ill-defined causes among the elderly in Brazil, 1979-2013: ecological study: Tendências de mortalidade por causas mal definidas em idosos no Brasil, 1979-2013: estudo ecológico

**DOI:** 10.1590/1516-3180.2016.0070010616

**Published:** 2016-09-26

**Authors:** Davi Félix Martins, Ridalva Dias Martins Felzemburg, Acácia Batista Dias, Tania Maria Costa, Pedro Nascimento Prates Santos

**Affiliations:** I MD. Assistant Professor, Department of Health, Universidade Estadual de Feira de Santana (UEFS), Feira de Santana, BA, Brazil.; II PhD. Adjunct Professor, Nursing School, Universidade Federal da Bahia (UFBA), Salvador, BA, Brazil.; III PhD. Adjunct Professor, Department of Humanities and Philosophy, Universidade Estadual de Feira de Santana (UEFS), Feira de Santana, BA, Brazil.; IV MD. Adjunct Professor, Department of Health, Universidade Estadual de Feira de Santana (UEFS), Feira de Santana, BA, Brazil.

**Keywords:** Death certificates, Mortality, Cause of death, Aged, Information systems, Atestado de óbito, Mortalidade, Causa de morte, Idoso, Sistemas de informação

## Abstract

**CONTEXT AND OBJECTIVE:**

Mortality measurements are traditionally used as health indicators and are useful in describing a population's health situation through reporting injuries that lead to death. The aim here was to analyze the temporal trend of proportional mortality from ill-defined causes (IDCs) among the elderly in Brazil from 1979 to 2013.

**DESIGN AND SETTING:**

Ecological study using data from the Mortality Information System of the Brazilian Ministry of Health.

**METHODS:**

The proportional mortality from IDCs among the elderly was calculated for each year of the study series (1979 to 2013) in Brazil, and the data were disaggregated according to sex and to the five geographical regions and states. To analyze time trends, simple linear regression coefficients were calculated.

**RESULTS:**

During the study period, there were 2,646,194 deaths from IDCs among the elderly, with a decreasing trend (ß -0.545; confidence interval, CI: -0.616 to -0.475; P < 0.000) for both males and females. This reduction was also observed in the macroregions and states, except for Amapá. The states in the northeastern region reported an average reduction of 80%.

**CONCLUSIONS:**

Mortality from IDCs among the elderly has decreased continuously since 1985, but at different rates among the different regions and states. Actions aimed at improving data records on death certificates need to be strengthened in order to continue the trend observed.

## RESUMO

**CONTEXTO E OBJETIVO:**

Medidas de mortalidade são tradicionalmente usadas como indicadores de saúde e são úteis na descrição da situação de saúde de uma população relatando lesões que levam à morte. O objetivo foi analisar a tendência temporal da mortalidade proporcional por causas mal definidas (CMD) em idosos no Brasil entre 1979 e 2013.

**DESENHO E LOCAL:**

Estudo ecológico utilizando dados do Sistema de Informação de Mortalidade do Ministério da Saúde no Brasil.

**MÉTODOS:**

A mortalidade proporcional por CMD em idosos foi calculada para cada ano da série estudada (1979 a 2013) no Brasil, e os dados foram desagregados por sexo e de acordo com as cinco regiões geográficas e estados. Foi empregado o coeficiente de regressão linear simples para analisar a tendência temporal.

**RESULTADOS:**

Durante o período de estudo, houve 2.646.194 mortes por CMD em idosos, com uma tendência decrescente (ß -0,545; intervalo de confiança, IC: -0,616 a -0,475; P < 0,000) em homens e mulheres. Essa redução também foi observada nas macrorregiões e estados, com exceção do Amapá. Os estados da região Nordeste registraram uma redução média de 80%.

**CONCLUSÕES:**

A mortalidade por causas mal definidas em idosos tem diminuído continuamente desde 1985 em ritmos distintos entre as regiões e estados. As ações destinadas a melhorar os registros de dados em certificados de óbito devem ser fortalecidas para dar continuidade na tendência observada.

## INTRODUCTION

Mortality measurements are traditionally used as health indicators^1^ and are useful in describing a population's health situation through reporting injuries that lead to death. This allows authorities, among other things, to prioritize the allocation of resources in accordance with the mortality profile identified.^2^ Mortality indicators assist in monitoring the trends of the most prevalent causes of death in a population and hence identify which segments are affected to a greater or lesser extent by certain diseases. The numerators of these indicators are obtained from the Mortality Information System (SIM) of the Ministry of Health, which also functions as a strategic tool for management of the healthcare system.^3^

Mortality statistics are only infrequently used. This is partly because of lack of completeness of the data fields that comprise death certificates (DCs), particularly the field representing the underlying cause of death. The lack of information in this field limits the explanatory power of death records regarding mortality patterns in a population.

In situations in which it is not possible to identify the underlying cause of death, such as lack of medical care, failure of doctors to properly maintain assignations and records or missing information, the cause of death is classified as an ill-defined cause (IDC). These correspond to the codes of Chapter XVIII (symptoms, signs and abnormal clinical and laboratory findings not elsewhere classified; codes R00-R99) of the 10^th^ Revision of the International Classification of Diseases (ICD-10), and Chapter XVI (symptoms, signs and ill-defined conditions; codes 780-799) of the 9^th^ Revision (ICD-9).

High proportions of reported deaths classified as ill-defined causes can significantly alter the mortality rates for specific diseases. This can distort a given community mortality profile and consequently reduce the potential use of these statistics for diagnosing the health of a given population and for planning and administering healthcare services for that population.^4^

By international standards, Brazil was characterized as having high levels of ill-defined causes of death in the middle 1990s.^5^ Over the last three decades, the Brazilian government has made significant investments that have improved vital registration systems over recent years.^6^^,^^7^ The completeness of death counts increased from 80% in 1980-1991 to 95% in 2000-2010, while at the same time the percentage of ill-defined causes of deaths was reduced by about 53% in the country, but with large regional differences. The south and southeast have much better data quality than the rest of the country.^8^

The distribution of ill-defined causes according to demographic characteristics, such as gender and age, is marked by higher incidence among men and the elderly (elderly is defined here as 60 years of age or older). Among the elderly, it is particularly difficult to identify the cause of death^9^ because of the presence of comorbidities (hypertension, diabetes, cancer, arteriosclerosis, dyspnea upon exertion, osteoarthritis and reduced visual acuity, among others) that frequently occur among the elderly. Moreover, age can influence the clinical expression of signs and symptoms,^10^ and it may be difficult to deal with the elderly, who may refuse to seek treatment and only do so in the later stages of the disease when there is greater impairment, which can hinder or even prevent establishment of diagnoses. Therefore, it is essential to monitor the quality of the information relating to the underlying cause of death among the elderly and the information relating to the demand for healthcare and social services, so as to better develop care planning and health promotion in this age group.^11^

Mortality trends can be identified from mortality rates in which the risk of death due to a specific cause is measured; or through proportional mortality, in which the relative importance of a disease or group of diseases is reported. In this study, we chose to work with proportional mortality to assess the weight of ill-defined causes of death among the elderly.

## OBJECTIVE

The objective of this study was to analyze the evolution of proportional mortality as a result of ill-defined causes of death among the elderly in Brazil during the period 1979-2013.

## METHODS

This ecological study used time series and exploratory analyses^2^ in which secondary data were used. All deaths registered as ill-defined causes among the elderly (detailed in Chapter XVI of ICD-9, for the period between 1979 and 1995; and in Chapter XVIII of ICD-10 from 1996 onwards) were included in this study. This system was implemented between 1975 and 1976, and the computerized database became available for viewing/capture on the web pages of the Information Technology Department of the Brazilian National Health System (DATASUS), with data from 1979 on.^12^

This study used data in the public domain. Thus, there was no need for approval from a research ethics committee. Two spatial scales were used for data analysis: Brazil and its macroregions (north, northeast, south, southeast and center-west). The proportional mortality due to IDCs among the elderly was calculated for each year of the study series (1979 to 2013) in Brazil, and the data were disaggregated according to sex and to the five geographical regions. The proportion of IDC deaths for each sex was calculated based on the total number of deaths for each sex.

Simple linear regression coefficients were calculated to examine the nature and significance of the temporal trend of proportional mortality from ill-defined causes among the elderly. In this analysis, the variable of time, expressed in years, was entered into the model as the independent variable, and the variable proportions of deaths from IDCs, overall and separated according to gender and geographical region, functioned as the dependent variables. To perform the data analysis, we used Tabwin,^13^ which is a public-domain spreadsheet provided by DATASUS, Ministry of Health; and the R software, which is a public-domain statistical package.

## RESULTS

**Figure 1:**
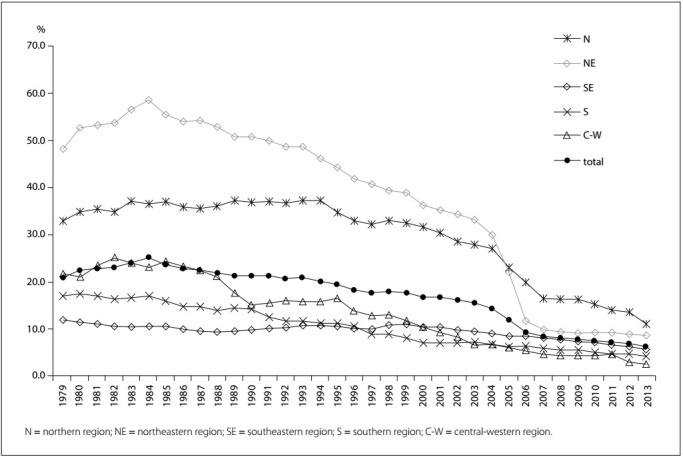
Proportion of deaths from ill-defined causes among the elderly, in relation to the total number of deaths recorded for the elderly, in Brazil overall and in its macroregions, 1979-2013.

During the study period, there were 2,646,194 deaths from ill-defined causes among the elderly, corresponding to an average of 75,606 deaths/year. The highest frequency of these deaths (883,162 deaths) occurred in the 1990s, accounting for 33.4% of the total number of deaths. Sex was not reported on 2,943 (0.11%) of the death certificates. There was a significant drop in the proportion of ill-defined causes of death among the elderly for Brazil overall, decreasing from 20.7% in 1979 to 6.2% in 2013 (Figure 1). The highest proportion (25.2%) was recorded in 1984: the underlying cause of death for a quarter of the elderly people who died in 1984 was not identified. In 2005, more than 10.0% of deaths among the elderly were from an unknown underlying cause.

The temporal trend of the proportional mortality from IDCs among the elderly decreased both for Brazil overall (ß: -0.545; confidence interval, CI: -0.616 to -0.475; P < 0.000) and for the macroregions. In the northeastern region, the proportional mortality from IDCs decreased from 48.1% in 1979 to 8.5% in 2013 (ß: -1.584; CI: -1.802 to -1.367; P < 0.000); in the central-western region, the proportional mortality from IDCs decreased from 21.7 to 2.6% (ß: -0.708; CI: -0.767 to -0.650; P < 0.000); in the northern region, the proportional mortality from IDCs decreased from 33.0 to 11.1% (ß: -0.725; CI: -0.874 to -0.576; P < 0.000); in the southeastern region, the proportional mortality from IDCs decreased from 11.9 to 5.7% (ß: -0.123; CI: -0.154 to -0.092; P < 0.000); and in the southern region, the proportional mortality from IDCs decreased from 17.1% in 1979 to 4.2% in 2013 (ß: -0.427; CI: -0.452 to -0.402; P < 0.000).

In the northeastern region, between the years 1980 and 1990, more than 50.0% of deaths among the elderly were classified as IDC, reaching a peak of 58.5% in 1984; this proportion was 5.6 times higher than that in the southeast (10.5%) in 1984. Since 1997, IDC deaths have accounted for less than 10.0% of the total number of deaths among the elderly in the south; this occurred ten years later in the northeast (in 2007).

In 2004, the northeastern region recorded the highest proportion of deaths from IDCs. In 2005, the highest proportion of deaths occurred in the northern region. This region was the only one to record a proportion greater than 10.0% in the last year of the study: in 2013, the proportion of deaths from IDCs among the elderly in the northern region was 11.1%, while the national average was 6.2%.

**Figure 2:**
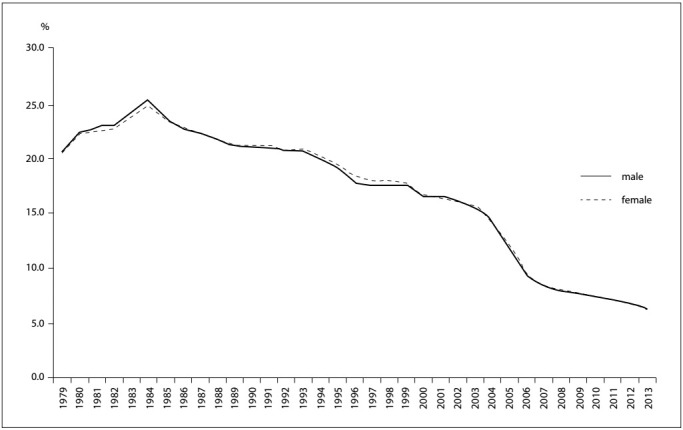
Proportion of deaths from ill-defined causes among the elderly according to sex, Brazil, 1979-2013.

The distribution according to sex showed similar patterns for men and women. The proportion of IDCs increased from 1979 until 1984, when it reached a peak of 25% for both sexes, followed by a decreasing trend extending until the last year of the study, when it reached 6.2%. The trend showed a decrease for both males (ß: -0.549; CI: -0.618 to -0.479; P < 0.000) and females (ß: -0.540; CI: -0.613 to -0.467; P < 0.000). Since 2006, the proportion of deaths due to IDCs among the elderly has remained below 10.0% in both sexes (Figure 2).

Table 1 shows that in two states, Roraima and Amapá, which are both located in the north, there was an increase in the proportion of deaths from IDCs among the elderly between the years 1996 (when ICD-10 started to be used) and the latest year for which mortality data were available on the DATASUS website. There were reductions in all the other states, particularly in the states of Tocantins in the northern region, Rio Grande do Norte in the northeastern region and Espírito Santo in the southeastern region, which had reductions of more than 90%. In 2013, only four states had rates that exceeded 10% (Amazonas, Pará and Amapá in the north and Bahia in the northeastern region).

**Table 1:**
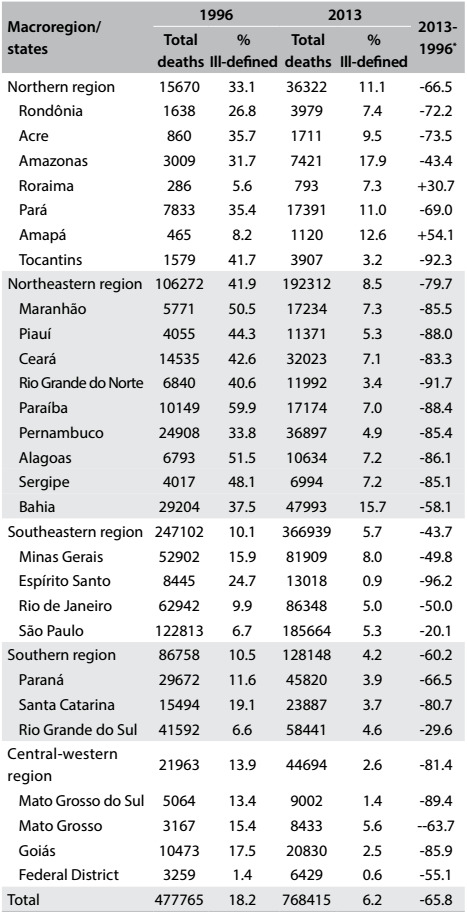
Proportional mortality from ill-defined causes among the elderly according to macroregions and states in selected years (1996 and 2013)

Source: Mortality Information System (SIM), Health Situation Analysis Department, Health Surveillance Secretariat, Ministry of Health. ^*^Percentage change 2013-1996 {(t_1_ - t_0_)/t_0_ *100}.

**Figure 3:**
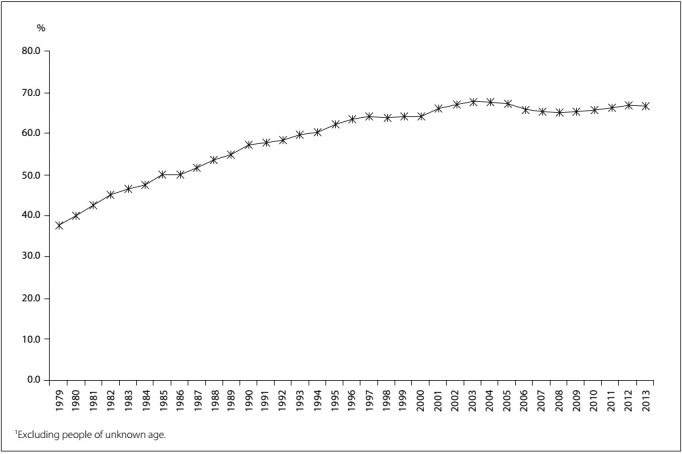
Proportion of deaths from ill-defined causes among the elderly in relation to the total number of deaths registered as having ill-defined causes,^1^ Brazil, 1979-2013.

The proportion of deaths from ill-defined causes among the elderly, within the total number of IDC deaths registered in Brazil during the period 1979-2013, displayed an upward trend with minor fluctuations, increasing from 37.8% in 1979 to 64.0% in 1997 and reaching 66.6% in 2013 (Figure 3).

## DISCUSSION

The time series analysis on the proportional mortality from ill-defined causes among the elderly revealed a marked and progressive decrease during the study period. This reduction was observed for the country as a whole, with regional variations that were accentuated in the north-south direction, ranging from a reduction of approximately 95.5% in the state of Espírito Santo, which is located in the southeastern region, to an increase of 147.8% in Amapá in the northern region. The largest decreases were observed in the states of the northeastern region, which reported an average decrease of more than 80.0%, except for the state of Bahia (60.4%).

The geographic variation in this indicator can be partially attributed to the different levels of economic development presented by the federal states, the care structure provided for the population, the structure and organization of health information record systems, the ease of access to healthcare services and the effects from improving qualification-oriented programs.

A study conducted in the state of São Paulo that evaluated health information^14^ identified significant regional health variations in relation to ill-defined causes of death among the elderly. The proportional mortality from ill-defined causes of death for the state in 2010 was 5.6%, ranging from 1.0% in the greater ABC region to 19.4% in the central DRS II region. The significant regional health differences observed in the state of São Paulo reflect deep inequalities in living conditions, which are closely related to social and economic factors and have a major influence on the quality of vital statistics records.^15^

Although occurrences of IDCs among the elderly have displayed a significant reduction, they remain high compared with other countries. In the period between 2002 and 2006, IDC deaths in the general population accounted for 1.5% of deaths in Colombia.^16^ In the United States, IDCs in the general population accounted for less than 2.0% of all deaths,^17^ and in 2003 in Chile, the percentage of deaths recorded under this classification was 2.8%.^18^ The pattern observed in these countries differs greatly from that found in South Africa, where the proportion of ill-defined causes of death increased from 12.2% in 1999 to 13.9% in 2007.^19^

Keer-Pontes and Rouquayrol^20^ stated that deaths from ill-defined causes reflect "not only worse quality of life and health of the population, but also lower quality or lack of medical care provided to that population". They also reported that in cases in which the deaths received a clinical follow-up in the final stages of the disease, poor completion of the DC was partly related to diagnostic errors as a result of lack of technical resources or personnel deficiencies, ignorance of the correct way to complete a DC, bureaucratic issues and attention to family prejudices against stigmatizing diseases (syphilis, AIDS and alcoholism).

Investigations that have assessed the quality of DC data show that the vast majority of deaths categorized as from ill-defined causes result from doctors incorrectly completing the DC.^9^ Adequately diagnosed cases are reported using terms that are vague or poorly defined, such as "cardiac arrest" and "multiple organ failure", which under coding rules are classified in Chapter XVIII of ICD-10.^9^

Since 2005, the Brazilian government has focused on helping states and municipalities from the poorest regions (north and northeast) through targeting increased completeness and reduction of ill-defined causes of death. This effort produced an important decrease in the proportion of ill-defined causes from all regions over time, especially in 2006‐2010, with improvements in terms of both magnitude and reduction of differentials across regions.^21^

The downward trend in the proportion of this type of death is widespread, but we believe that peculiarities are maintained in some age groups such as the elderly. The high occurrence of home deaths in this age group^9^ also increases the chance of the death being classified as having an ill-defined cause. A study conducted in four state capitals that assessed mortality from ill-defined causes among the elderly from 1996 to 2007 found that in Porto Alegre, 50% of deaths classified as IDCs occurred at home.^22^ In the state of São Paulo in 2010, 48.2% of these events occurred at home, and only 44.2% occurred in hospitals or other healthcare facilities. Moreover, unattended deaths, which are one of the most common situations for ill-defined causes of death, represented 31% of all ill-defined causes of death. These deaths occur predominantly at home (42.6%).^14^

Moreover, the classification of deaths did not differ according to sex. The downward trend in both sexes suggests that the factors that caused this reduction produced the same effects in both sexes.

The high proportions of IDC deaths observed in the 1980s may reflect a time when the need for services and healthcare professionals to assist the population was greatest. This need resulted from uneven spatial distribution of healthcare services and professionals in Brazil, which were primarily located in the southern and southeastern regions and in major urban centers.

The above mentioned problems added to the difficulty of the population's access to healthcare services and the organization of health surveillance services.^23^ The disabilities of the organization of health surveillance services depicts a situation of serious neglect of a problem regarding information that was more marked that decade, thus reflecting a failure to comply with the mandatory registration of vital events, and specifically deaths, and the lack of importance given to this by those in charge of planning healthcare actions.

The household living situations of the elderly, whether in rural or urban areas, may explain the resistance to or greater difficulty in health service provision. The predominantly rural location pattern probably affects the demand for healthcare because rural populations have less access to and therefore make less use of healthcare services.^24^^,^^25^ This may result partly from transportation difficulties, financial constraints and greater resistance to seeking medical care.

We believe that seeking medical care was a more important issue in the mid-1980s. A significant proportion of the population over 60 years of age during that decade came from a cohort that was primarily born in rural areas, and a fraction of that population is still alive today. Brazil was historically a country with predominantly rural characteristics, but since the 1950s, Brazil has been undergoing a transformation into a more urbanized country. Only in the 1960s did the urban population exceed the rural population.^26^ The low frequency of healthcare services use among people living in rural areas throughout their lives is a behavioral characteristic that may change as these people become older, given that the demand for healthcare services can be expected to increase. This increase in the frequency of healthcare services use among the elderly has been previously demonstrated. This stage of life is characterized by a greater biological vulnerability associated with higher prevalence of diseases and disabilities.^27^

The cohorts born in the 1930s and subsequently differed from those that preceded them in relation to household status at birth. Some of those who were born in the countryside migrated into cities, and this pattern became more pronounced in the 1950s and 1960s, and extended into the early 1990s. According to the Brazilian Institute for Geography and Statistics (IBGE) (2001),^26^ the proportion of the population living in urban areas increased from 67.5% in 1980 to 75.5% in 1991 and 81.2% in 2000, partly because of the intense rural-urban migration process.

This population has had more experience with urban standards in relation to healthcare and seeking treatment. This new social integration has resulted in increased demand for healthcare and has coincided with a period of intensifying public campaigns addressing various healthcare service issues, such as the Elderly Vaccination Campaign, in which people aged 65 year and older were immunized against influenza, beginning in 1999. In 2000, the campaign began offering immunization to those over 60 years old. Campaigns towards the elderly may have contributed towards encouraging them to seek healthcare services, thus reducing the culturally constructed resistance.

The availability of healthcare services and professionals prepared to meet the needs of this contingent, which swells the population, is a challenge for both the state and society. Populations have the ability to extend their average lifespan and therefore age is an indicator of social evolution, which is influenced by the pattern of economic development and the technical/scientific attainment level of the society to which the population belongs. Such achievements are a source of concern for both society and the state, which need to adjust to new demands, and they have an impact on the economic and social structure. The challenge ahead for the twenty-first century is to provide quality-of-life support to a growing elderly population of primarily low socioeconomic and educational level and high prevalence of chronic diseases and disabilities.^28^^,^^29^

Apart from these unwieldy problems, the reduction in the proportion of ill-defined causes of death points towards the possibility of achieving an even lower level if the ongoing actions are intensified. The Brazilian states with the worst indicators need to be prioritized, so as to identify the main causes of the poor quality of information and implement a series of actions to reverse this situation. Among the measures that could help reduce occurrences of deaths categorized as having ill-defined causes is continuous monitoring of what is causing death in this age group, with training and skill transfers for municipalities with greater difficulties, with the aim of reaching physicians in these regions and encouraging them to fill out the underlying cause of death on the death certificate. 

This study was based on large spatial units, including macroregions and states, for the data analysis. Although this allowed approximation of occurrences and the spatial distribution of the event analyzed, the spatial unit size can be considered to be a limitation of this study. Use of smaller spatial units, such as regional health districts or even municipalities, would provide knowledge in greater detail, including identification of the localities with major problems in classifying deaths, and would allow interventions to be targeted to the most deprived locations.

## CONCLUSIONS 

Proportional mortality from ill-defined causes among the elderly was seen to present a marked progressive decrease during the study period. This reduction was observed for the country as a whole, with regional variations accentuated in the north-south direction. These variations require geographically differentiated interventions in order to reduce their occurrence. Thus, improving the quality of mortality statistics among the elderly is essential in order to provide valid and reliable data for producing information to support healthcare planning for this group of elderly individuals.
